# Takotsubo Cardiomyopathy Complicated by High-Grade Atrioventricular Block in a Patient With Preexisting Conduction Disease: A Case Report

**DOI:** 10.7759/cureus.98876

**Published:** 2025-12-10

**Authors:** Nicholas G Pitto, Kirollos Philops, Kokhoon Tay, Louise A Brown

**Affiliations:** 1 Cardiology, Portsmouth Hospitals University NHS Trust, Portsmouth, GBR; 2 Cardiology, Multidisciplinary Cardiovascular Research Centre, Leeds Institute of Cardiovascular and Metabolic Medicine, University of Leeds, Leeds, GBR; 3 Biomedical Imaging Science Department, Leeds Institute of Cardiovascular and Metabolic Medicine, University of Leeds, Leeds, GBR

**Keywords:** cardiac resynchronization therapy (crt), high degree av block, permanent pacemaker implantation (ppm), takotsubo cardioyopathy, trifascicular block

## Abstract

Takotsubo cardiomyopathy (TTC) often mimics acute coronary syndrome, presenting with chest pain, electrocardiogram (ECG) changes, and troponin rise. While usually reversible, it can rarely be complicated by conduction abnormalities such as high-grade atrioventricular (AV) block.

We report a 79-year-old woman with poorly controlled hypertension and preexisting trifascicular block who presented with chest pain precipitated by extreme emotional stress due to the inability to obtain her antihypertensive tablets prescription via her General Practitioner, coinciding with the anniversary of her husband’s death. Initial evaluation based on her 12-lead ECG was in keeping with a lateral ST elevation myocardial infarction, but emergency invasive coronary angiography revealed unobstructed coronaries with classical apical ballooning of the left ventricle. Transthoracic echocardiography (TTE) and cardiac magnetic resonance imaging (MRI) within 48 hours confirmed TTC. Over the following days, she developed Mobitz II AV block progressing to high-grade AV block. Given her persistent poor left ventricular systolic function and high risk of pacing dependency, the consensus opinion after discussion in the multidisciplinary device team meeting opted for a cardiac resynchronisation therapy pacemaker (CRT-P), which was implanted successfully.

This case highlights the rare association between TTC and high-grade AV block, particularly in patients with underlying conduction disease. Early recognition and individualised pacing strategies are essential for improving outcomes.

## Introduction

Takotsubo syndrome (TTC), first described in the 1990s in Japan [1], can often present similarly to an acute coronary syndrome, with chest pain, raised cardiac enzymes and ECG changes. It is named after the characteristic left ventricular changes, in the absence of coronary artery disease, which resemble Japanese octopus traps. It disproportionately affects older women; about 90% of 1750 cases in the International Takotsubo Registry were women with a mean age of 67 [1]. Whilst previously thought of as being triggered by emotional stressors, both positive and negative, physical stressors such as physical activities and medical conditions or procedures are more common triggers and nearly one third occur in the absence of any known trigger [1-2]. Although the pathophysiology of TTC remains poorly understood, it is thought to be associated with sympathetic stimulation and catecholamine excess [1].

There are cases of high-grade AV block as a complication of TTC in the literature [3-5] as well as reports of CHB [6-8]. A review of arrhythmia occurrence with TCC looked at 1876 patients and found 2.9% had AV node dysfunction, including Mobitz type I AV block, Mobitz II AV block and complete heart block [9]. Below, we present a case of a patient with preexisting trifascicular block with TTS who went on to develop high-grade AV block and required device therapy.

## Case presentation

**Figure 1:**
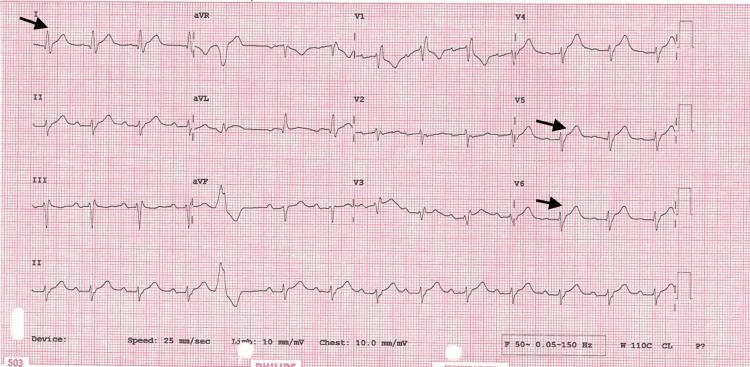
Admission ECG showing trifascicular block and ST elevation (arrows)

**Figure 2:**
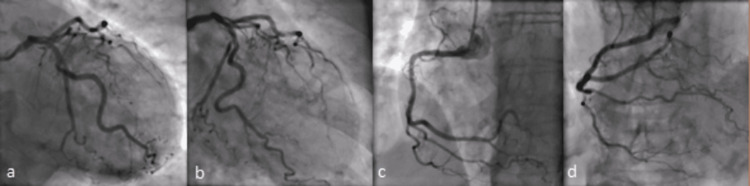
Angiogram showing unobstructed coronary arteries, a) PA caudal, b) RAO caudal, c) LAO RCA, d) PA cranial RCA LAO RCA: Right coronary angiogram in left anterior oblique; PA: Posteroanterior

**Figure 3:**
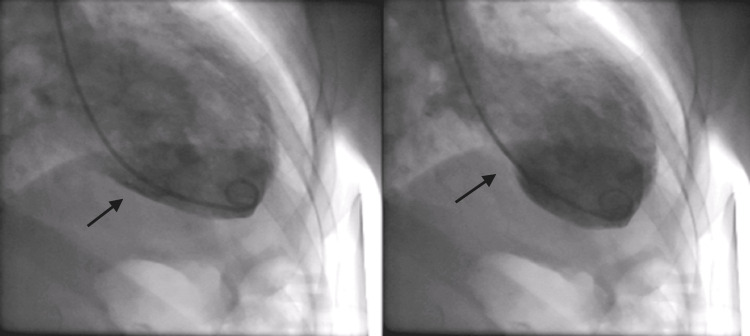
Ventriculography showing apical ballooning (arrows) in diastole (left) and systole (right).

A 79-year-old woman with a background of poorly controlled hypertension presented with severe central crushing chest pain at rest. This occurred during a period of emotional stress on the anniversary of her husband's death and coinciding with being unable to obtain her antihypertensive medications from her general practitioner. On presentation to the emergency department, her admission ECG was suggestive of lateral myocardial infarction (MI), as well as trifascicular block with a prolonged PR interval, left axis deviation and right bundle branch block (RBBB) and some premature ventricular ectopics (Figure 1). Her initial troponin-T was elevated at 165 ng/L [0-11 ng/L] and then rose to 690 ng/L. She was taken to the cardiac catheterisation laboratory as an emergency case for primary percutaneous coronary intervention (PPCI). Her angiogram, however, showed non-obstructed coronaries (Figure 2), but ventriculography showed poor left ventricular (LV) function, with akinesia of the distal segments of her left ventricle and a pattern typical of TTC (Figure 3).

Her echocardiogram showed severe left ventricular systolic dysfunction, with a left ventricular ejection fraction (LVEF) of ~35%, a moderately dilated left ventricle and impaired diastolic function. Her left atrium was also severely dilated (Video 1). She was started on guideline-directed medical therapy for her heart failure, namely dapagliflozin, sacubitril/valsartan, bisoprolol and eplerenone.

**Video 1:** TTE showing EF~35%, dilated left ventricle and mid to apical akinesis EF: Ejection fraction; TTE: Transthoracic echocardiography

**Figure 4:**
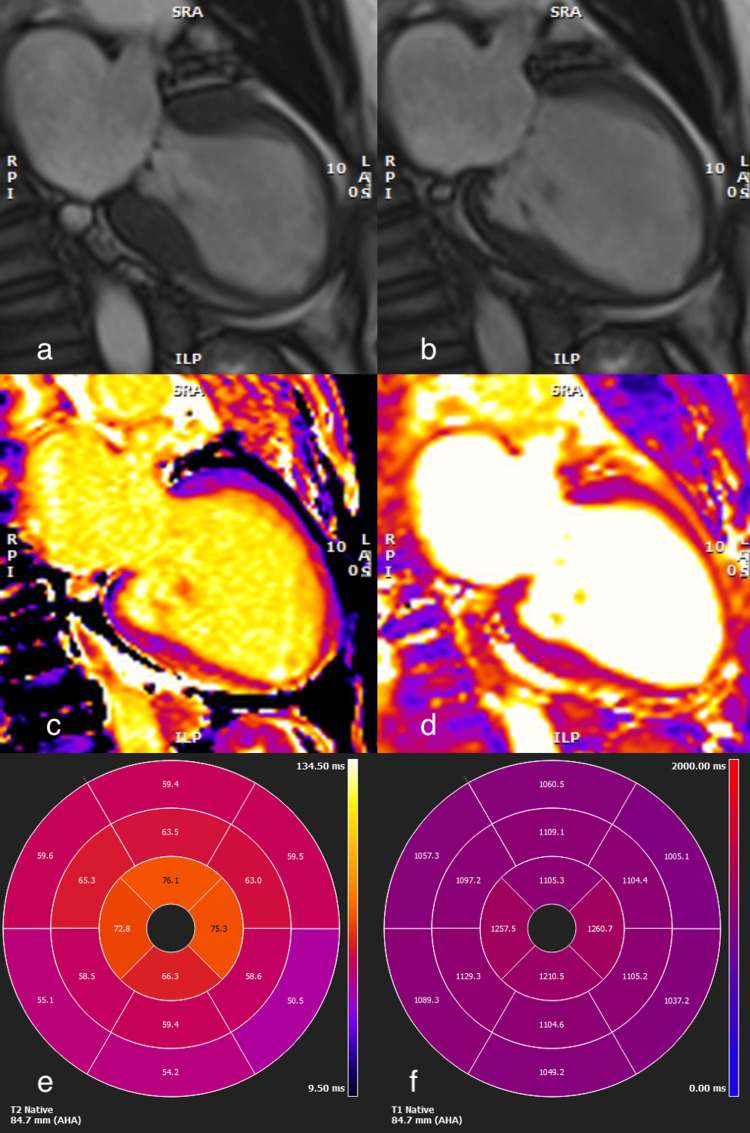
Cardiac MRI images showing dilated LV in Takotsubo-like pattern a) 2-chamber systole, b) 2-chamber diastole, c) T1 2-chamber, d) T2 2-chamber. Bullseye plots showing 16 segment distribution of e) T2 and f) T1 values, with a typical distribution as seen in Takotsubo (elevated from mid ventricle to apical levels). LV: Left ventricular

**Figure 5:**
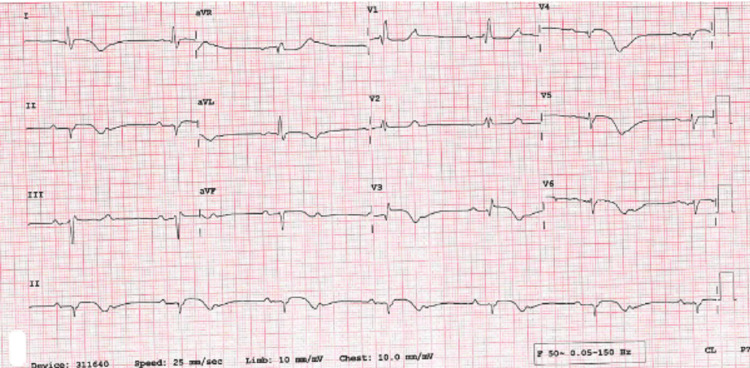
ECG showing 2:1 AV block AV: Atrioventricular

**Figure 6:**
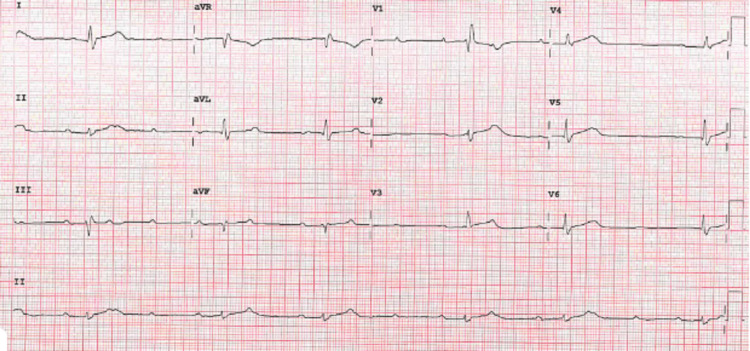
ECG showing high-grade AV block AV: Atrioventricular

As a case of myocardial infarction with non-obstructed coronary arteries (MINOCA), she went on to have cardiac MRI 48 hours post admission, which showed a dilated left ventricle with severely reduced ventricular function in mid to apical myocardium in a Takotsubo-like pattern. T1 and T2 levels were elevated in midventricular and apical myocardium in keeping with near transmural oedema and inflammation (Figure 4). Overall, these findings, together with the clinical history, were suggestive of Takotsubo cardiomyopathy. After her MRI, she was noted to be in a 2:1 AV block (Figure 5). Four days later, she had a repeat echocardiogram, which did not show any significant changes compared to the initial one. At this time, she was noted to be in high-grade AV block (Figure 6).

Due to the persistent high-grade AV block, discussions were held in the device multidisciplinary team meeting, and the consensus was that, due to the high-grade AV block [10], pre-existing trifascicular block and poor LVEF, she would benefit from a CRT-P, which was implanted without immediate complications two days later. Aside from a postoperative haematoma and a slight derangement in her renal function, which improved back to baseline, she was discharged several days later. 

## Discussion

TTC is a transient heart failure syndrome that often follows emotional or physical stress. It can look very similar to an acute coronary syndrome, with chest pain, ECG changes, and a rise in cardiac enzymes. It is characterised by the recovery of LV function in the majority, if not all, patients, with data from the large multicentre InterTAK Registry led by Templin and colleagues showing that almost all patients regain normal left ventricular function [11]. In a small number, complications such as arrhythmias or high-grade atrioventricular block can develop. When TTC and conduction disease occur together, it can be difficult to determine whether one caused the other or if they simply coexist.

From a diagnostic perspective, TTC is increasingly recognised as a dynamic process that can be identified early through a combination of ECG and echocardiography. Although the initial ECG may mimic an anterior ST elevation myocardial infarction (STEMI), emerging analyses have shown patterns of electromechanical mismatch that can help differentiate TTC from left anterior descending (LAD) territory infarction. Recent work by Borkowski et al. [12] introduced the LAD Electromechanical Mismatch Index, comparing ECG findings with regional longitudinal strain. Their results showed that patients with TTC demonstrate discordance between ECG severity and the degree of segmental LV dysfunction on strain imaging, unlike LAD STEMI, where both align. This supports the growing role of advanced echocardiographic techniques-particularly speckle-tracking strain-in recognising the characteristic ballooning patterns and ruling out ischemic causes. Even conventional echocardiography remains central in diagnosis, as apical or mid-ventricular ballooning, hyperkinesis of the basal segments, and rapid improvement on repeat scans are key hallmarks of TTC.

In this case, the patient’s emotional stress, normal coronary angiogram, and the classic pattern of apical ballooning on ventriculography were all consistent with TTC. Her later progression from Mobitz type II block to high-grade AV block was significant, especially given her background of trifascicular block. The likely explanation is that myocardial swelling or temporary ischemia in the conduction system, triggered by the catecholamine surge seen in TTC, worsened her existing conduction problem and led to high-grade AV block.

The incidence of AV block in TTC is low, reported around 2.9% [9], but recovery varies. Small series suggest that around one-third to one-half regain AV conduction as ventricular function recovers, while the rest remain pacing-dependent, often due to underlying disease. Several case reports have described TTC unmasking pre-existing trifascicular or His-Purkinje disease, supporting the idea that catecholamine-related myocardial stunning can tip borderline conduction systems into complete block. These cases mirror the interaction seen in our patient and reinforce how TTC can worsen conduction abnormalities even when the LV dysfunction itself is expected to recover.

This makes the case educational because it reinforces the need to consider TTC in patients who unexpectedly deteriorate in their conduction status.

Previous case reports have described similar interactions between TTC and underlying conduction disease, including trifascicular block, where transient myocardial dysfunction appears to unmask or worsen pre-existing conduction abnormalities. This makes the case educational because it reinforces the need to consider TTC in patients who unexpectedly deteriorate in their conduction status.

When managing such patients, the key decision is whether AV conduction will recover or if pacing is needed. Some cases of TTC-related high-grade AV block resolve once the ventricular function improves, but others remain dependent on pacing. In this patient, the presence of poor LVEF (35%), established conduction disease, and the high risk of long-term pacing dependency supported the decision to implant a cardiac resynchronisation therapy pacemaker (CRT-P) rather than a standard device. This follows current European and Heart Rhythm Society pacing guidance, which recommends CRT-P instead of right ventricular pacing in patients with reduced ejection fraction or anticipated high pacing burden to maintain ventricular synchrony and reduce pacing-related deterioration. This option was chosen to maintain ventricular synchrony and reduce the risk of pacing-related heart failure.

## Conclusions

This case highlights the importance of considering TTC as a potential cause of advanced conduction disease. Early recognition, careful monitoring, and multidisciplinary input are essential. Individualised pacing decisions can help achieve the best possible outcomes, particularly in older patients with baseline conduction abnormalities or reduced ventricular function. The take-home message is that TTC can coexist with and worsen underlying conduction disease, and clinicians should remain aware that high-grade AV block may develop, particularly in older patients or those with known conduction abnormalities. Early recognition, multi-disciplinary team input, and guideline-based pacing decisions are essential.
